# Population-based palliative care planning in Ireland: how many people will live and die with serious illness to 2046?

**DOI:** 10.12688/hrbopenres.12975.1

**Published:** 2019-12-03

**Authors:** Peter May, Bridget M. Johnston, Charles Normand, Irene J. Higginson, Rose Anne Kenny, Karen Ryan

**Affiliations:** 1 Centre for Health Policy and Management, Trinity College Dublin, Dublin, Dublin, D2, Ireland; 2 The Irish Longitudinal study on Ageing, Trinity College Dublin, Dublin, Dublin, D2, Ireland; 3 Cicely Saunders Institute of Palliative Care, Policy and Rehabilitation,, King's College London, London, SE5 9PJ, UK; 4 Palliative Medicine, Mater Misericordiae University Hospital, Dublin, D07 R2WY, Ireland; 5 School of Medicine, University College Dublin, Belfield, Dublin, D04 V1W8, Ireland

**Keywords:** palliative care, terminal care, multimorbidity, quality of life, utilization, epidemiology, mortality

## Abstract

**Background:** All countries face growing demand for palliative care services.  Projections of need are essential to plan care in an era of demographic change.   We aim to estimate palliative care needs in Ireland from 2016 to 2046.

**Methods: **Static modelling of secondary data.  First, we estimate the numbers of people in Ireland who will die from a disease associated with palliative care need.  We combine government statistics on cause of death (2007-2015) and projected mortality (2016-2046).  Second, we combine these statistics with survey data to estimate numbers of people aged 50+ living and dying with diseases associated with palliative care need.  Third, we use these projections and survey data to estimate disability burden, pain prevalence and health care utilisation among people aged 50+ living and dying with serious medical illness.

**Results:** In 2016, the number of people dying annually from a disease indicating palliative care need was estimated as 22,806, and the number of people not in the last year of life aged 50+ with a relevant diagnosis was estimated as 290,185.  Equivalent estimates for 2046 are 40,355 and 548,105, increases of 84% and 89% respectively.  These groups account disproportionately for disability burden, pain prevalence and health care use among older people, meaning that population health burdens and health care use will increase significantly in the next three decades.

**Conclusion: **The global population is ageing, although significant differences in intensity of ageing can be seen between countries. Prevalence of palliative care need in Ireland will nearly double over 30 years, reflecting Ireland’s relatively young population.  People living with a serious disease outnumber those in the last year of life by approximately 12:1, necessitating implementation of integrated palliative care across the disease trajectory. Urgent steps on funding, workforce development and service provision are required to address these challenges.

## Introduction

### Background

Population ageing reflects significant advances in medicine and wider society, but increasing longevity brings new health care challenges in all countries. More people are living longer with serious illness, multimorbidity and functional impairment, creating an urgent need to develop workforce and grow service capacity ^1, 2^. This is not only a question of upscaling existing services but reconfiguring systems originally designed to provide acute, episodic treatment ^3^.

Palliative care is an approach that aims to improve pain and symptom management, communication and planning in care of people with serious medical illness ^4^. Palliative care may be provided by specialists, whose core activity is palliative care, and generalists, who are not engaged full time in palliative care but have some relevant training that allows them to practice a palliative care approach as part of usual service provision ^5^.

Originating in end-of-life care, palliative care approaches are now widely seen as having potential benefits across the trajectory of serious disease including improved quality of life and, in some circumstances, extended survival ^6– 8^. The appropriate mix of specialist and generalist involvement in care may be determined by the persistence and complexity of needs, with greater specialist involvement for patients with more complex needs ^9^. Some services, notably bereavement support, focus predominantly on the end-of-life and post-death period. Palliative care access is highly variable internationally, and under-supply and unmet need are widely reported even in countries with relatively strong provision ^10, 11^.

A 2017 study by Etkind *et al.* used cause-of-death data to project palliative care need in England and Wales to 2040 ^12^. They estimated that the number of deaths will increase 25% in a 25-year period, and the number of deaths from a disease indicating palliative care need (e.g. cancer, organ failure, Alzheimer’s and related dementias) will rise 25–43%, depending on projection method. Expected increases are due to population ageing and growing prevalence of serious chronic disease, with the biggest effects observed in those aged 85+. The United Kingdom has an old population relative to the European Union average ^13^. Countries with younger populations, irrespective of high-, middle- and low-income status, can expect faster rates of growth in palliative care need ^1^.

### Rationale and aim

All countries face inadequate current supply and growing future demand for palliative care services. Accurate projections of future need are essential to inform expansion of current services and development of new models of care in an era of demographic change. Quantifying deaths from a disease indicating palliative care need is a critical first step. Equivalent cause-of-death projections for Ireland to those made in England and Wales do not currently exist, and this study was funded by a Health Service Executive Healthy Ageing Award to address the Irish evidence gap. We detail our methods and data in full to assist researchers in replicating and extending our approach in their own countries.

It is also important to move beyond estimations of need that are based solely on cause of death. This is because cause of death data recorded on death certificates are often subjective where the decedent had one or more condition ^14, 15^, and risk undercounting palliative care needs if the cause of death does not indicate palliative care need but concurrent unrecorded conditions do. More significantly, policy and clinical guidelines increasingly recommend palliative care across the trajectory of serious disease, meaning that those dying from these diseases are only a subset of the overall population health need ^6, 16^. To inform decision-making in policy and practice, including the targeting of interventions to those who benefit, groups cannot be defined by their characteristics at death and instead must be identified prospectively on the basis of clinical and other relevant factors ^17, 18^.

We aim to project future palliative care needs in the Irish setting. First, we replicate the Etkind *et al.* (2017) methodology with Irish cause-of-death data. We combine two sources of publicly available government statistics – recorded death registry data 2007–2015, and population projections 2016–2046 – to estimate numbers of people dying from a disease indicating palliative care need in Ireland to 2046. Second, we combine official population projections with survey data to estimate to 2046 numbers of people aged 50+ living and dying with diseases indicating palliative care need. Third, we use these projections and survey data to estimate disability burden, pain prevalence and health care utilisation among people aged 50+ living and dying with diseases indicating palliative care need. These are respectively labelled Analysis 1, Analysis 2 and Analysis 3 throughout.

## Methods

### Design

 Secondary research study of already-collected data. In Analysis 1, we used routinely accessible statutory data in Ireland on recorded deaths 2007–2015, and projected mortality 2016–2046. In Analyses 2 and 3, we retained projected mortality data, and combined these with projected population data 2016–2046 and observed individual-level data from a prospective longitudinal study on ageing.

### Setting

Ireland has a relatively young population among high-income countries but faces typical trends in global ageing ^19^. Palliative care services are well established by international standards ^20^ and since 2001 are recognised by a national policy recommending universal provision on the basis of need ^21^. However, some aspects of policy are yet to be implemented resulting in gaps in access to aspects of generalist and specialist services in all parts of the country ^22^. This is in the context of variable access for non-palliative care services: a means-tested medical card grants free primary and hospital care, and subsidized prescription medicines; people without a medical card pay capped co-payments for hospital care and prescriptions, and full primary care costs out of pocket ^23^. Ireland has the second highest level of self-reported unmet needs for specific health care-related services due to financial reasons in the European Union ^24^.

Studies have shown that decedents and their close family members still experience suboptimal outcomes that include unmanaged pain and depression, and place of death inconsistent with their preferences ^25, 26^. An update of the policy is expected to start in 2020 ^27^. This will take place in the wider context of Sláintecare reforms, a wide-ranging set of recommendations to embed universal entitlements into the Irish health and social care system including palliative care access ^28^.

### Data sources

In Analysis 1, we used only data routinely available from the statutory Central Statistics Office (CSO) in Ireland. For recorded causes of death, we accessed the most recently available all-cause mortality data. These were for the period 2007 to 2015. For estimated number of future deaths in Ireland, we accessed the most recently available mortality projections by age and gender. These were for the period 2016–2046.

In Analyses 2 and 3, we retained mortality projections (2016–2046), and we accessed the CSO’s total population projections for the same period. We also accessed data from The Irish Longitudinal study on Ageing (TILDA). TILDA is a biannual survey of adults aged 50+ living in Ireland. Details of sampling, interviews, health assessments, response rates and calibration weights have been described previously ^29– 31^. Briefly, TILDA Wave 1 (2009–2011) recruited a representative sample of community-dwelling adults aged 50+ in the Republic of Ireland. Information is collected on a wide range of topics including health, financial, social and family circumstances, and use of health and social care services. At Wave 1 each participant completed a computer-assisted personal interviews (CAPI) and self-completion questionnaires (SCQ), and a trained nurse conducted a comprehensive health assessment ^29– 31^. Subsequent waves are biannual (Wave 2 in 2012, Wave 3 in 2014, etc). CAPI and SCQ follow-up occurs at each Wave; health assessments were conducted at Wave 3 and are planned again at Wave 6 in 2020. In the event of a TILDA participant’s death, a family member or close friend is approached to complete a voluntary interview on their end-of-life experience ^25^. The end-of-life interview covers demographics; disability and level of assistance; physical, behavioural and mental health; and health and social care utilisation and assets; and complements equivalent sections of regular TILDA participant interviews ^32^. This study uses data from Wave 3 (2014) to estimate exposure and outcome variables among older people living in Ireland, and end-of-life interviews Waves 1–5 to estimate exposure and outcome variables in the last year of life.

All CSO projections were published in 2013. All CSO data are available to download from their website ^33^ and we include all relevant data sheets alongside our calculations in *Underlying/extended* data ^34^. We also present aggregate TILDA data by age and gender in *Underlying/extended* data ^34^. To protect anonymity, TILDA does not report cell sizes under 20; where necessary we pooled sub-groups that were defined by gender and five-year age band in order to meet this n=20 threshold for all reported data.

### Exposure variables: diseases indicating palliative care need

In Analysis 1, we classified each death in mortality records (2007–2015) as from a disease indicating palliative care need [0|1] using International Classification of Disease (ICD) codes and those groupings established previously by Etkind *et al.* (2017) ( Table 1) ^12^.

**Table 1.** ICD-10 codes for cause of death to identify palliative care need. Reprinted from Etkind *et al*. (2017) ^12^ under a Creative Commons Attribution 4.0 International License.

	ICD-10 code	Conditions
**Cancer**	C00–C97	Malignant neoplasms
**Organ failure**	I00–I52 (excl. I12 & I13) J40–J47, J96 I12, I13, N17, N18, N28 K70–K77	Heart disease, heart failure, chronic lower respiratory disease, respiratory failure, reno-vascular disease, renal failure, liver disease
**Dementia**	F01, F03, G30, R54	Dementia, vascular dementia, Alzheimer’s disease, senility
**Other**	G10, G12.2, G20, G23.1, G35, G90.3 I60–I69 B20–B24	Huntington’s disease, motor neurone disease, Parkinson’s disease, progressive supranuclear palsy, multiple sclerosis, multi-system atrophy; haemorrhagic, ischaemic and unspecified stroke; HIV

In Analyses 2 and 3, we classified each Wave 3 TILDA participant as living with a disease indicating palliative care need [0|1] if they had reported a diagnosis of at least one of the following conditions: cancer, heart attack, congestive heart failure, chronic lung disease such as chronic bronchitis or emphysema, chronic kidney disease, serious liver disease or cirrhosis, dementia, Alzheimer’s disease, Parkinson’s disease or stroke. We classified each TILDA decedent for whom end-of-life interview data were collected as dying with a disease indicating palliative care need [0|1] if they had reported any of these diagnoses in a CAPI prior to death, or if the end-of-life interview with a friend or family member reported any such diagnosis. The following conditions listed in Table 1 are not asked in TILDA, although respondents always have the opportunity to self-report specific conditions: Huntington’s disease, motor neurone disease, progressive supranuclear palsy, multiple sclerosis, HIV. For further details on concordance between CSO codes and TILDA diagnoses data, and a quantification of arising under-estimation of relevant disease prevalence using TILDA data, see *Underlying/extended data:* 20191101 Appendix TILDA Prevalence ^34^.

### Outcome variables: disability burden, pain prevalence and health care use

In Analyses 2 and 3, we calculated for each TILDA participant their outcomes as follows:

- **Disability burden** using the Activities of Daily Living (ADL) index ^35^. These deficits are identified by asking which activities the participant required help with: dressing, crossing a room, bathing, eating, getting in/out of bed, toileting. Each participant therefore has a score in the range 0–6 based on the number of deficits.
- **Pain burden** using responses to questions on pain severity, ‘Are you often in pain?’ [Yes/No] and ‘How bad is the pain most of the time?’ [Mild/Moderate/Severe]. We attached values to responses so that 0=not regularly troubled by pain; 0.25=mild pain; 0.5=moderate pain and 1=severe pain.
- **Health and social care use.** General practitioner (GP) visits, emergency department (ED) admissions, overnight acute hospital inpatient admissions and hours of home help received were collected as frequency data: how many times was each category used in the last 12 months.

Each of these outcomes was calculated for all TILDA Wave 3 participants, and for all TILDA decedents to Wave 5 with an end-of-life interview.

### Projection methods

***Analysis 1: estimated total number of people in Ireland dying from a disease indicating palliative care need to 2046.*** We replicated the Etkind *et al.* (2017) approach to estimate future palliative care need in England and Wales: ^12^

- **Method 1:** Gomez-Batiste calculation ^36^. An estimated 75% of people who die, do so with palliative care needs. We access the total number of projected deaths annually in Ireland to 2046, and calculate 75% of this number.
- **Method 2a:** Constant need. The proportion of deaths annually with palliative care need is estimated as % of all deaths with palliative care need at baseline. The most recent year for population cause of death data was 2015. We calculated proportion of 2015 deaths from a relevant disease and combined this proportion with the total number of projected deaths annually in Ireland to 2046.
- **Method 2b:** Assuming annual change, prior eight years. The proportion identified in **2a** is not assumed to be constant but, rather, increases according to a compound interest rate, *r*, which is calculated as mean rate of annual change over the prior nine years (i.e., 2007 to 2015 inclusive).
- **Method 2c:** Assuming annual change, prior three years. A replication of **2b**, except that *r* is calculated as mean rate of annual change over the prior four years (i.e., 2012 to 2015 inclusive).
- **Method 2d:** Assuming annual change, by age and gender. A replication of **2b**, except that *r* is calculated by gender for each five-year age band.

***Analysis 2: estimated total number of people aged 50+ in Ireland living and dying with a disease indicating palliative care need to 2046.*** We estimated the number of people dying in Ireland in a given year, by age and gender, from the CSO mortality projections (2016–2046). We estimated the number of people alive in Ireland throughout a given year, by age and gender, by subtracting mortality projections from CSO population projections (2016–2046). We calculated the proportion of people aged 50+ living with a disease indicating palliative care need, by age and gender, in TILDA Wave 3. We calculated the proportion of people aged 50+ dying with a disease indicating palliative care need, by age, in TILDA end-of-life interviews. For each year, we multiplied the number of people projected by the CSO to live through the year by the proportion in TILDA living with a disease indicating palliative care need, and we multiplied the number of people projected by the CSO to die by the proportion in TILDA dying with a disease indicating palliative care need. Step-by-step calculations can be seen in *Underlying/extended data* ^34^.

***Analysis 3: estimated outcomes among people aged 50+ in Ireland living and dying with a disease indicating palliative care need to 2046.*** For each outcome of interest (disability burden, pain prevalence, utilisation categories), we calculated the mean, adjusted for age, gender and palliative care disease, among people aged 50+ in TILDA Wave 3 and end-of-life interviews. We combined these calculations with the population projections from Analysis 2.

To quantify growth in disability and pain burden, and use of different health care services, in a single comprehensible index we set values in each outcome in 2016 to 100. We then created indices to 2046 with this 2016 value as a base (so a 50% increase in any outcome to 2046 gives a 2046 score of 150, etc.). Health service utilisation is therefore based on current patterns, implicitly assuming no changes in policy or access during the projection period. Step-by-step calculations can be seen in *Underlying/extended data* ^34^.

## Results

### Analysis 1: estimated total number of people in Ireland dying from a disease indicating palliative care need to 2046

**Figure 1.**
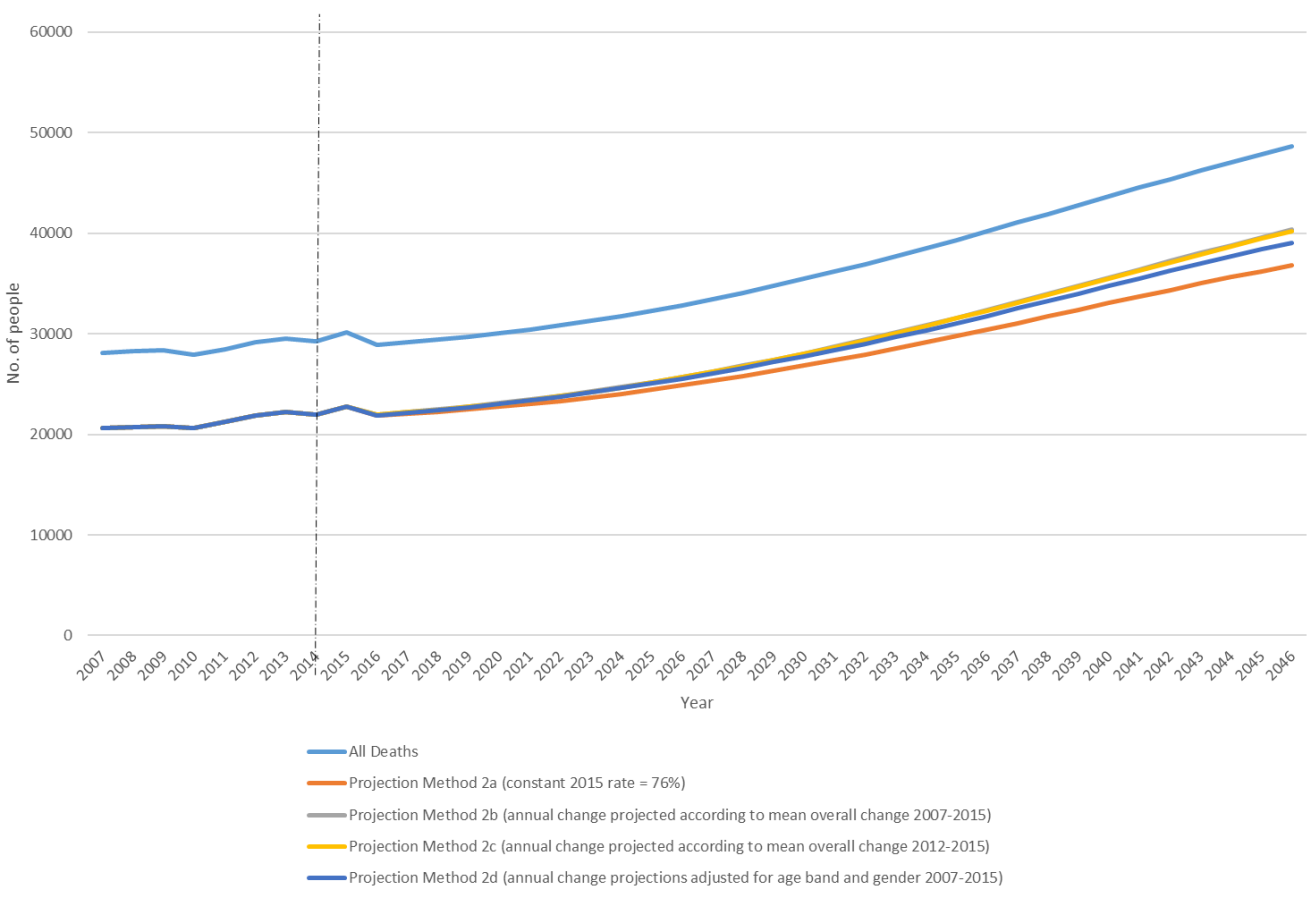
Estimated number of deaths in Ireland from a disease associated with palliative care need 2016–2046, by projection method.

Total number of projected deaths, and estimated proportions of these deaths from a disease associated with palliative care need, are presented in Table 2 and Figure 1.

**Table 2.** Observed and projected total deaths, and proportions from a disease associated with palliative care need, in Ireland to 2046.

		2015	2026	2036	2046
**All recorded deaths**	*N _y_=*	30,127			
**Deaths recorded with PC need**	*n _y_=* *p(N _y_)=*	22,806 76%			
***All projected deaths***	*N _y_=* *Rate of change index=*		32,860 114	40,209 139	48,631 168
***Deaths projected with PC need***					
*Method 1: Gomez-Batiste, 75% of all deaths*	*n _y_=* *p(N _y_)=* *Rate of change index=*		24,645 75% 114	30,157 75% 139	36,473 75% 168
*Method 2a: Constant needs from 2015, 76% of all deaths*	*n _y_=* *p(N _y_)=* *Rate of change index=*		24,875 76% 114	30,438 76% 139	36,813 76% 168
*Method 2b: Annual change, 2007–2015*	*n _y_=* *p(N _y_)=* *Rate of change index=*		25,699 78% 117	32,392 81% 147	40,355 83% 184
*Method 2c: Annual change, 2012–2015*	*n _y_=* *p(N _y_)=* *Rate of change index=*		25,666 78% 117	32,314 80% 147	40,211 83% 183
*Method 2d: Annual change, 2007–2015, by age/gender*	*n _y_=* *p(N _y_)=* *Rate of change index=*		25,554 78% 117	31,796 79% 145	39,081 80% 178

For full details of each projection method, see Methods>Projection methods. PC=palliative care. *N _y_*=total number, all deaths in a given year (y); *n _y_*=number of deaths from a disease indicating palliative care need according to a given projection method; *p*( *N _y_*) is proportion of deaths in a given year with PC need, i.e. ( *n _y_*/ *N _y_*)*100; Rate of change index=(n _y_/[n _2016_])*100

Total number of recorded deaths at baseline in 2015 was 30,127. Total number of projected deaths in 2046 was 48,631. This is a 30-year increase of 68%. By definition, methods using constant need (Methods 1 and 2a) mirror this growth: 68% increase in number of deaths from a disease associated with palliative care need to 2046.

Using any method that incorporated changing needs (2b, 2c, 2d), total number of projected deaths from a disease associated with palliative care need in 2046 ranged from 39,081 (=80% of all deaths in 2046; method 2d) to 40,355 (=83% of all deaths of all deaths in 2046, method 2b). These represent a 2016–2046 increase in absolute numbers of deaths from a disease associated with palliative care need between 78% and 84%.

**Figure 2.**
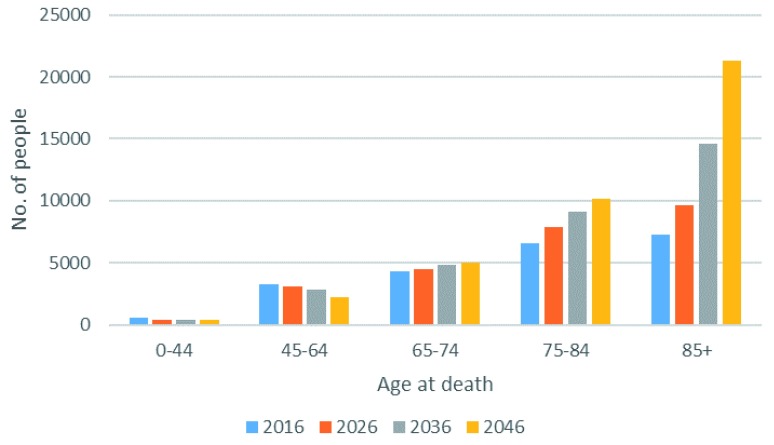
Estimated number of deaths in Ireland from a disease associated with palliative care need 2016–2046, by age.

Projected numbers of deaths from a disease associated with palliative care need by age are presented in Figure 2. Both absolute numbers of these deaths at each time point, and the projected growth in numbers over time, are heavily driven by the oldest age groups (85 years and over).

### Analysis 2: estimated total number of people aged 50+ in Ireland living and dying with a disease indicating palliative care need to 2046

**Figure 3.**
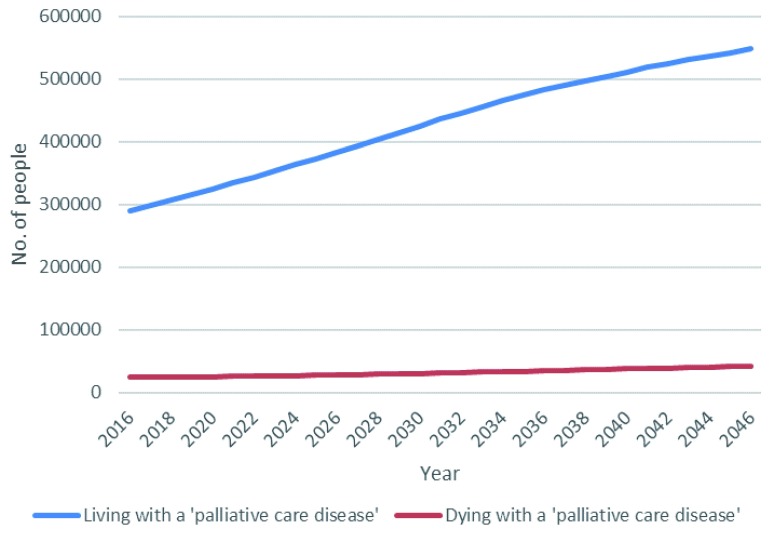
Estimated number of people aged 50+ in Ireland living and dying with a disease associated with palliative care need 2016–2046.

Projected numbers of people aged 50+ in Ireland to 2046, living and dying with diseases associated with palliative care, are presented in Table 3 and Figure 3.

**Table 3.** Estimated numbers aged 50+, living and dying with a disease associated with palliative care need, in Ireland to 2046.

		2016	2026	2036	2046
*Total population aged 50+ in Ireland*	*N _y_=* *Rate of change index=*	1,428,313 -	1,818,264 127	2,181,617 153	2,292,217 160
*Living with a disease associated* *with palliative care need*	*n _y_=* *p(N _y_)=* *Rate of change index=*	290,185 20% -	383,675 21% 132	482,548 22% 166	548,105 24% 189
*Living without a disease associated* *with palliative care need*	*n _y_=* *p(N _y_)=* *Rate of change index=*	1,111,200 78% -	1,403,530 77% 126	1,661,105 76% 149	1,698,751 74% 153
*Dying with a disease associated with* *palliative care need*	*n _y_=* *p(N _y_)=* *Rate of change index=*	24,257 2% -	28,145 2% 116	34,859 2% 144	42,193 2% 174
*Dying without a disease associated* *with palliative care need*	*n _y_=* *p(N _y_)=* *Rate of change index=*	2,672 <0.5% -	2,914 <0.5% 109	3,105 <0.5% 116	3,168 <0.5% 119

For full details of palliative care need, see Methods>Exposure variables. ‘Living with(out)’ = live the entirety of a given year; ‘Dying with(out)’ = die within a given year; *N _y_*=total population aged 50+ in Ireland in a given year (y); *n _y_*=number for a given sub-group in a given year; *p*( *N _y_*) = sub-group as a proportion of 50+ population, i.e. ( *n _y_*/ *N _y_*)*100; Rate of change index=(n _y_/[n _2016_])*100

Total population of people aged 50+ in Ireland between 2016 and 2046 was estimated to rise from 1,428,313 to 2,292,217, an increase of 60%. The number of older people living through the year with a palliative care need was estimated to grow from 290,185 to 548,105, an increase of 89%. This group accounted for 20% of the older population in 2016, rising to 24% by 2046. The number of older people dying annually with a palliative care need was estimated to grow from 24,257 to 42,193, an increase of 74%. This group accounted consistently for about 2% of the population aged 50 and over across the timeframe of analysis.

### Analysis 3: estimated outcomes among people aged 50+ in Ireland living and dying with a disease indicating palliative care need to 2046

**Figure 4.**
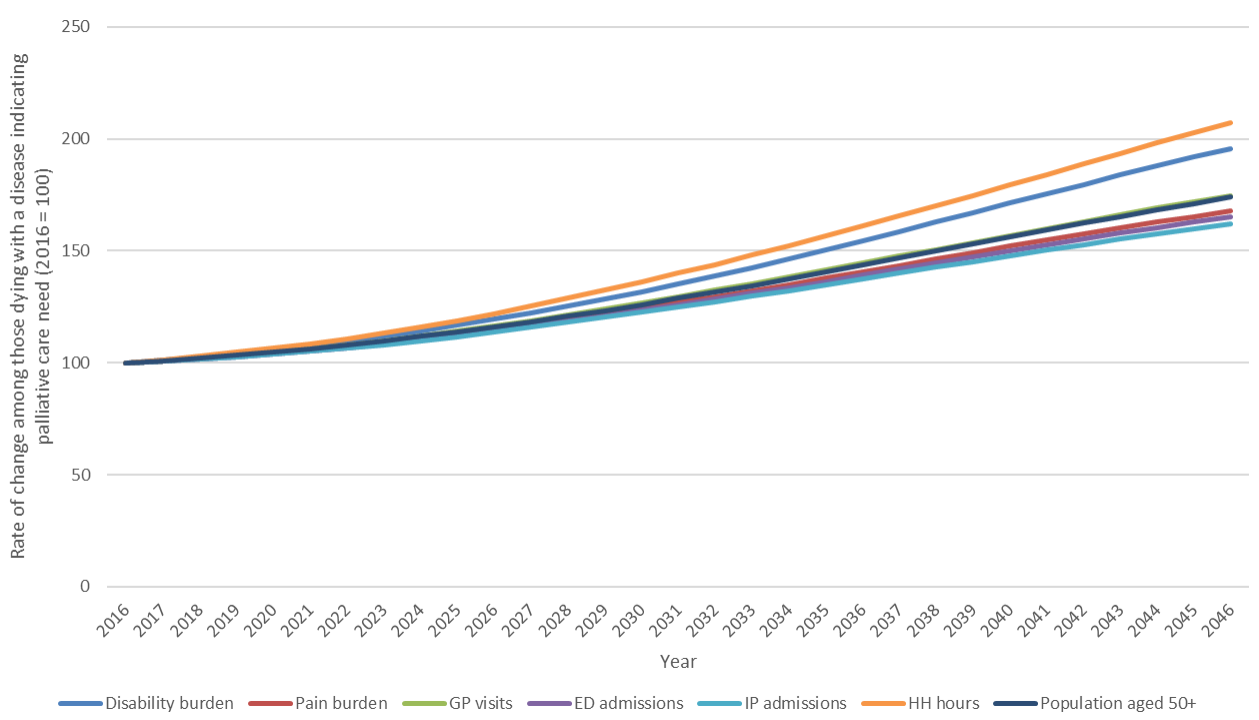
Estimated pain prevalence, disability burden and health care use among people aged 50+ in Ireland dying with a disease associated with palliative care need 2016–2046.

Estimated increases in outcomes for older people dying with a disease indicating palliative care need are presented in Figure 4. Disability burden was projected to increase 96% between 2016 and 2046, and pain burden is projected to increase 68%. Large increases in health care use were also predicted for all categories: GP visits (74%), emergency department admissions (65%), inpatient hospital admissions (62%) and home health hours (107%).

**Figure 5.**
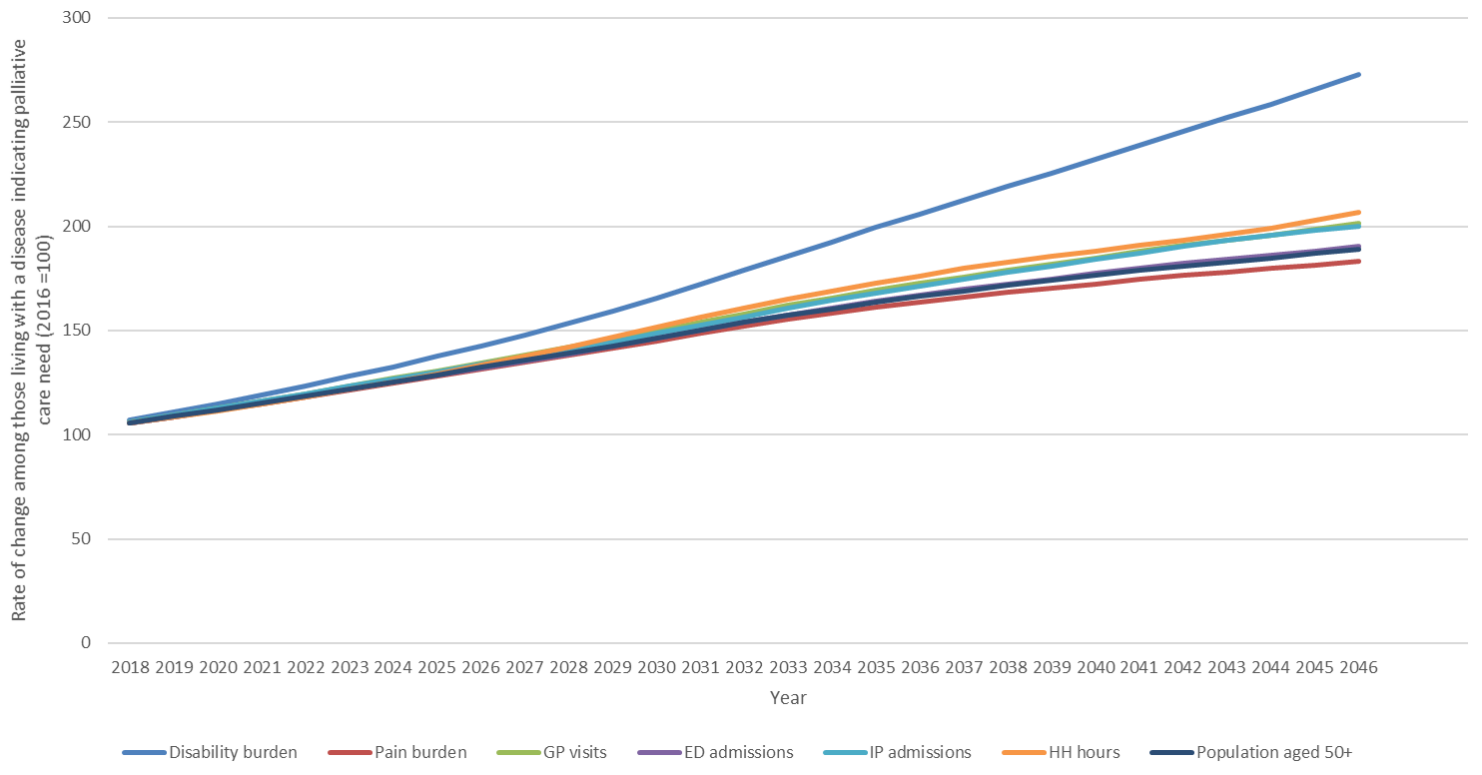
Estimated pain prevalence, disability burden and health care use among people aged 50+ in Ireland living with a disease associated with palliative care need 2016–2046.

Estimated increases in outcomes for older people living through the year with a disease indicating palliative care need is presented in Figure 5. Disability burden is projected to increase 173% between 2016 and 2046, and pain burden is projected to increase 83%. Large increases in health care use are also predicted for all categories: GP visits (101%), emergency department admissions (90%), inpatient hospital admissions (100%) and home health hours (106%).

The proportion of estimated outcomes accounted for by those living and dying with diseases indicating palliative care are presented in Table 4.

**Table 4.** Estimated outcomes among people living and dying with palliative care-relevant diseases aged 50+ in Ireland to 2046, as a proportion of total outcomes among those aged 50+.

	2016	2026	2036	2046
**Living with a disease associated with** **palliative care need**				
* Proportion of population aged 50+*	20%	21%	22%	24%
* Disability burden*	39%	42%	44%	46%
* Pain burden*	25%	26%	27%	28%
* GP visits*	29%	30%	32%	33%
* ED admissions*	37%	39%	39%	39%
* Inpatient admissions*	34%	35%	36%	37%
* Home health hours*	46%	45%	42%	38%
**Dying with a disease indicating** **palliative care need**				
* Proportion of population aged 50+*	2%	2%	2%	2%
* Disability burden*	25%	22%	21%	21%
* Pain burden*	3%	3%	3%	3%
* GP visits*	5%	4%	4%	5%
* ED admissions*	15%	13%	12%	12%
* Inpatient admissions*	10%	9%	9%	10%
* Home health hours*	11%	10%	9%	9%

For full details of each outcome, see Methods>Outcome variables. ‘Living with’ = live the entirety of a given year; ‘Dying with’ = die within a given year; all data points represent the outcome total in a given year for a given population as a proportion of total outcome in all people aged 50+ in Ireland.

People in the last year of life with a palliative care need account heavily for observed outcomes. This group was 2% of those aged 50+ in 2016, yet they are estimated to account for 25% of the disability burden and 3% of the pain burden. Under current patterns of service use, they also account for 5% of GP visits, 15% of ED admissions, 10% of inpatient admissions and 11% of home help hours. Assuming no change in patterns of use, these proportions are fairly consistent to 2046, as this group remains 2% of all people aged 50+.

People living with a palliative care need also account disproportionately for outcomes, although this disproportionality is not so pronounced. This group was 20% of those aged 50+ in 2016, yet they are estimated to account for 39% of the disability burden, 25% of the pain burden, 29% of GP visits, 37% of ED admissions, 34% of inpatient admissions and 46% of home help hours. All proportions increase moderately to 2046, as this group increases to 24% of all people aged 50+.

## Discussion

### Key results

The number of people in Ireland dying from a disease associated with palliative care need is estimated to increase 68–84% between 2016 and 2046, depending on projection method employed ( Table 2, Figure 1). The equivalent estimates for England and Wales between 2015 and 2040 are 25–43% ^12^. The difference primarily reflects Ireland’s relatively young population. Projected increases are underpinned by two factors: increasing absolute numbers of people dying (68% increase, 2016–2046), and increasing proportion of all deaths that are from a disease indicating palliative care need (from 76% in 2016 to 80–83% in 2046) ( Table 2). Increases are most observable in the 85+ age group ( Figure 2).

The number of people living with a disease associated with palliative care need outnumber those in the last year of life with a relevant diagnosis by 12:1, and this ratio is projected to remain fairly constant as both groups increase in absolute size over the next 30 years ( Table 3, Figure 3). People living and dying with a disease associated with palliative care need account disproportionately for disability burden, pain prevalence and health care utilisation ( Table 4). Per person burden is highest among those who are in the last year of life; total burden is larger among those with life expectancy of more than a year because they are a much larger group. Both groups, and so their associated health burdens and care needs, will increase significantly in size to 2046 ( Figure 4, Figure 5).

### Limitations

All projections use static modelling to extrapolate future trends. In prevalence projections we implicitly assume no change in disease profile by age and gender, but long-term changes are expected; e.g. smoking prevalence is on the decline so in the future older people should have fewer smoking-related problems than the current cohort, obesity prevalence is on the rise so future cohorts will have more related problems, future disability burden may grow more slowly than disease prevalence due to assistive technologies ^37^. Palliative care need is defined by prevalence of specific conditions but broader definitions of eligibility, e.g. to include multimorbidity ^38^, frailty ^39^ and pain prevalence ^40^, could be defended and would deliver larger projected increases ^12^. Future research may wish to revisit the definition of palliative care need in routine data ^41^ and longitudinal ageing surveys ^42^, particularly among the oldest old for whom a single diagnosis is not typically the best indicator of need.

In outcome projections we implicitly assume no exogenous changes in these; e.g. reduced pain through improved prescribing policies, increased service use due to ageing among unpaid family carers, reduced hospital use through health service reconfiguration ^28^. Health care use is estimated based on past use not need, so unmet need may be uncounted and unnecessary use over-counted. Dynamic modelling that takes account of the interaction of different variables over time, changing health profiles across generations and an evolving policy landscape, as well as cross-validating predictive accuracy and quantifying the uncertainty associated with all stages of projections, would offer a more sophisticated picture of future population numbers and their associated outcomes. Such analyses are planned.

All CSO population projections are based on assumptions of future fertility (F) and net migration (M) ^43^; our estimates rely on the CSO’s mid-range assumptions ‘F2’ (total fertility rate to decrease to 1.8 by 2026 and to remain constant thereafter) and ‘M2’ (net migration returning to positive by 2018 and rising slowly thereafter to plus 10,000 by 2021). Since deaths occur overwhelmingly among older age groups, and fertility and immigration impact demographics mainly among younger people, our headline conclusions are robust to alternative assumptions.

In Analysis 1, all data are reliant on death certificates whose reliability as a source of death is sub-optimal and variable ^14, 15^. In Analyses 2 and 3, disease prevalence and outcome data were available only for people aged 50+. While this group accounts for the large majority of people living and dying with diseases relevant to palliative care, there are also needs in younger groups that future analyses must address.

TILDA uses self-report data in regular waves, and proxy end-of-life interviews, both of which are subject to biases ^44, 45^. TILDA does not ask specifically about all causes of death listed in Table 1, risking under-estimate of relevant deaths. We reviewed CSO cause-of-death data, counting only diseases recorded by TILDA, and found a 1% discrepancy ( *Underlying/extended data:* 20191101 Appendix TILDA prevalence ^34^), so we believe this risk to be low. However, neurological diseases are usually associated with high burden of disability so future work should investigate this potential undercounting further. TILDA does not ask about pain specifically associated with terminal illness, so reported pain burden is as a proportion of all chronic pain self-reported by older people.

Analysis 1 quantifies people dying from a given set of diseases, Analyses 2 and 3 people dying with those diseases. The former are a subset of the latter. Our estimated 74% increase in those dying with a relevant disease ( Table 3) is around the midpoint of our 68–84% estimate for those dying from a relevant disease ( Table 1). Possible explanations include (i) Etkind *et al.* (2017) projection methods 2b, 2c, 2d for changing need overestimate future growth, inflating the Table 1 estimates; (ii) TILDA cell size restrictions forced us to pool age bands in the end-of-life interviews to 50–79 and 80+, potentially underestimating disease prevalence in some sub-groups and deflating Table 3 estimates. Future work can examine this further.

### Interpretation and policy implications

Where some high-income countries have already experienced their fastest rates of population ageing, this period is still approaching for Ireland. The rate of increase for people dying from a disease indicating palliative care need between 2016 and 2046 is estimated at 68–84%, compared to 25–43% for England and Wales (2015–2040). Those in their last year of life in Ireland with a disease with palliative care needs account heavily disproportionately for disability and pain burden, and health and social care use, and in many cases will require expert supportive care. Need for bereavement services and appropriate supports among family members will also grow commensurately. Ireland is far from unique in these demographic trends: European Union countries with faster rates of ageing include France, Belgium, the Netherlands, Czech Republic and Poland ^13^, while most low- and middle-income countries face challenges of a much larger magnitude ^1^. The World Health Organization’s Public Health Strategy for Palliative Care recommends that countries address all four components of the public health model when developing a whole systems response ^46^, and the response of individual countries to these demographic challenges can be tracked in documents such as the European Atlas for Palliative Care ^20^. While previous studies have predominantly used mortality data to estimate palliative care need, this study makes an original contribution to the literature by considering also estimations of palliative care need among those living with life-limiting illness. It is a notable finding that in any given year in Ireland, people aged over 50 living with a disease associated with palliative care need outnumber those in their last year of life with such a disease by approximately 12:1. While a small proportion of those living with relevant diseases require specialist care throughout their disease trajectory, many will benefit from some specialist palliative care input. Although occasional or episodic involvement of specialist palliative care services is proposed as the best practice model of care for Ireland ^47^, significant barriers to providing integrated palliative care exist ^48^. Focused efforts will be required to overcome these obstacles.

Notably the disability burden – as defined by the number of everyday tasks people require help with – will nearly treble among older people in the next 30 years unless novel treatment modalities are associated with improved disability-free survival rates. Lack of appropriate supports in everyday tasks is an established risk for health deterioration and avoidable service use ^39, 49, 50^. Reconfiguring care provision and medical training for an age of multimorbidity and complexity is critical to the needs of this group being met. System-wide projections suggest large increases in demand for services in all sectors even under optimistic assumptions around healthy ageing and service reconfiguration ^51^.

These trends are imposing, particularly given the challenges already facing the Irish health service, but those responsible for planning health care services are forewarned. Previous studies have found that both specialist and generalist palliative care capacity and funding in Ireland are well short of levels required to meet current need ^22, 52^. Universal access to generalist and specialist services will require multiplicative increases in available budgets ^28, 52^. Resource allocation is necessary but not the sole requirement to meet this growing demand. One foremost supply-side challenge is the identification, training and retention of staff to provide services on a scale outlined in our analyses, although this challenge is faced by the whole health care system. While workforce planning and development is always a complex task, there are particular issues related to palliative care services. For example, healthcare professionals have previously reported lacking the skills or confidence required to provide palliative care ^53, 54^. These deficits have been linked primarily to historical gaps in education and ongoing training in palliative care ^47^. However, variation in knowledge or experience in Ireland may also be attributable to regional differences in service provision ^55^.

Sláintecare project milestones for 2019 include the development of a revised national palliative care policy ^56^. Ireland is recognised to have a high standard of palliative care provision ^10, 11, 20^ and previous policy has done much to enable this. However, it is timely to reflect on the observation that historically key policy goals in palliative care were not realised in Ireland because of “large resource commitments required; the competition for resources from other, better-established healthcare sectors; and challenges in expanding workforce and capacity” ^52^. There is a growing literature focused on policy implementation that recognises that policies do not succeed or fail on their own merits, and that endeavours to close the policy-implementation gap. Chief among strategies recommended is improving policy design ^57^. The process of policy design should include the use of a good quality evidence base and the detailed population-focused analysis of need presented in this paper aims to serve this purpose in order to advance palliative care service development in Ireland.

## Conclusion

Annual deaths in Ireland from a disease associated with palliative care need are estimated to increase up to 84% between 2016 and 2046. The number of people in a given year living through the year with a disease indicating palliative care need is estimated to increase 89% in the same period. These increases are large compared to many other high-income countries, and reflect Ireland’s relatively young population that is in the early stages of demographic ageing. People living with a disease indicating palliative care need outnumber those in the last year of life with such a diagnosis by about 12:1, and this ratio is steady across the period of analyses. To meet population health needs requires urgent strategic steps on funding, workforce development and service provision in specialist and generalist palliative care. It is hoped that these data will be of value in informing the upcoming review of Irish national palliative care policy.

## Data availability

### Underlying and extended data

Open Science Framework: Appendix to: [Population-based projections of future palliative care need in Ireland], https://doi.org/10.17605/OSF.IO/53BJH ^34^

This project contains the following underlying/extended data:

- 20191101 Appendix Analysis 1 (All source data and results for Analysis 1)
- 20191101 Appendix Analyses 2 3 (All source data and results for Analyses 2 and 3)
- 20191101 Appendix TILDA Prevalence (Calculation of potential underestimation of palliative care need due to TILDA not explicitly recording some relevant diseases)

Data are available under the terms of the Creative Commons Attribution-ShareAlike 4.0 International license (CC BY-SA 4.0).

All CSO data can be downloaded directly from their website (last accessed October 17 ^th^, 2019): https://www.cso.ie/en/media/csoie/releasespublications/documents/population/2013/PLFP20162046ZipfileExcel.zip

TILDA data can be accessed in two ways. Access to all data, including end-of-life interviews, is available only from the TILDA servers at Trinity College Dublin. Application for access can be made via their website: https://tilda.tcd.ie/data/accessing-data/. Access to a reduced, harmonized version of Waves 1–4 can be accessed on application here: https://www.ucd.ie/issda/data/tilda/. TILDA recognizes replicability as an important part of science and on application can make available all .do files and data used in this study.
