# Supplementary material for: Population-based palliative care planning in Ireland: how many people will live and die with serious illness to 2046?
Source: HRB Open Res. 2019 Dec 3;2:35. [Version 1] doi: 10.12688/hrbopenres.12975.1 (PMC7017420; doi:10.12688/hrbopenres.12975.1)
Supplement: Peer review history

# Peer review: Reviewer response for version 1

**DOI:** 10.21956/hrbopenres.14060.r26992

**Published:** 2020-01-15

**Authors:** Anne Finucane

**Affiliations:** 1 Marie Curie Hospice Edinburgh, Edinburgh, UK

This paper describes findings from a study to project future estimates palliative care need in Ireland up to 2046.  It replicates an established methodology to estimate needs using national datasets. This study also extends previous work by estimating the number of people aged 50+ living and dying with diseases associated with palliative care need alongside disability burden, pain prevalence and health care utilisation.  This latter analysis is original and the provides important information on palliative care needs that has not previously been reported.  Some further detail and cross-checking of a small number of calculations is needed. However, overall the paper provides clear evidence for significant future increases in the number of people living and dying with palliative care needs in Ireland; and describes the likely impact on health care service use. This is vital information for service commissioners and policy-makers involved in decision-making about end-of-life care in Ireland.

**Abstract**

- Clear, comprehensive, well written.
- For those outside of Ireland, perhaps clarify if data refers to the island of Ireland or Republic of Ireland.

 

**Introduction**

 Please clarify whether the study is specific to the Republic of Ireland or the whole island of Ireland?  What is the population and number of deaths in recent year(s)? Is it possible to add a comparison graph comparing age distribution of the Irish population (current and projected if available) with that of the EU or other relevant comparison countries (e.g. England/Wales; Scotland).  This might provide useful detail on the extent to which the age profile of Ireland’s population differs from other high-income countries. This could be presented in the Introduction, or in the Methods (setting) section.

**Methods**

- Data sources: Please reference data sources used in the first paragraph under Data sources. 
- Perhaps clarify what are ‘exposure’ variables where they are first mentioned.
- Please add content to reference 34 link – further details could not be accessed. 
- Please check page 4, last paragraph – last sentence – should this say ICD-10 codes instead of CSO codes?
- In analysis 2 and 3, please justify why those *living *with cancer (as opposed to those who died with cancer) can be classified as having a disease associated with palliative care need, given increased survivorship in those with a cancer diagnosis.
- Please add detail on step-by-step calculations for Analysis 2 & 3 – reference 34 contains no data.

 

**Results**

- Please recheck percentages in Results, paragraph 2.  A 30-year increase of 68% is reported.  Is this correct?  Should this be 61% over 31 years? Please clarify.  
- Please provide detail on how the increase in absolute numbers of deaths from a disease associated with palliative care need was calculated (78% to 84%) as the percentages are not presented in the table. Please recheck all percentages reported. Is 84% correct?
- Very interesting data in Table 3 and related figures, with important implications for policy and service commissioning. 

 

**Discussion**

- Please check first line which states that the number of people in Ireland dying from a disease associated with palliative care need is estimated to increase between 68% to 84%.   However in the results section, end of paragraph 3, the percentages mentioned are 78% to 84%.  Please clarify.
- Please double-check Etkind *et al. *study for equivalent estimates for England and Wales (43% or 47% in Etkind *et al*?).
- Please elaborate on how Ireland’s younger population might account for the greater increases in palliative care need in Ireland compared with England and Wales.  This is not totally clear given that most of the increases in both studies comes from increases in the number of people dying aged 85+
- Limitations – last paragraph – should Table 1 read Table 2?
- “ *and that endeavours to close the policy-implementation gap*”. Is there something missing from this sentence?
- Important points for future approaches to estimating future need are described and will be helpful to other researchers working in this area.

**Conclusion**

- As above, please recheck data (84% increase?). 
- Overall the conclusion summarises the findings well and highlights important implications for palliative care policy and service delivery in Ireland.  

I confirm that I have read this submission and believe that I have an appropriate level of expertise to confirm that it is of an acceptable scientific standard, however I have significant reservations, as outlined above.

# Response

**Published:** 2020-01-21

**Authors:** Peter May

**Affiliations:** Trinity College Dublin, Ireland

We are grateful to the reviewer for their detailed and thoughtful report.  We have made a number of changes to the manuscript following these comments.  The most substantive change is the addition of a new Figure comparing Ireland’s population age distribution to the rest of the European Union (Figure 1, version 2).  We also add clarifying text on Ireland’s population ageing and the interpretation of our main results tables.

For the most part changes were minor.  Each specific change is documented in responses to the relevant reviewer report.  For each reviewer comment requiring action, we use the notation [R1/C1, R1/C2, etc], where R indicates which reviewer and C numbers each comment.  We mark our responses to each comment >>>.

**R2/C1** For those outside of Ireland, perhaps clarify if data refers to the island of Ireland or Republic of Ireland.

>>>Abstract changed to Republic of Ireland to avoid confusion. 

>>>In the main manuscript we continue to use ‘Ireland’ and provide the following explanation under Methods>Setting:

[Ireland] is sometimes called the Republic of Ireland to distinguish the sovereign state from the island of Ireland (incorporating Northern Ireland), but the country’s official name is Ireland and we use this form throughout.

**R2/C2** Please clarify whether the study is specific to the Republic of Ireland or the whole island of Ireland? 

>>>See R2/C1.

**R2/C3** What is the population and number of deaths in recent year(s)?

>>>Added to Methods>Setting

In 2015, the baseline year for our data, the estimated population was 4.7million people with 30,127 (0.006%) recorded deaths.

**R2/C4** Is it possible to add a comparison graph comparing age distribution of the Irish population (current and projected if available) with that of the EU or other relevant comparison countries (e.g. England/Wales; Scotland).  This might provide useful detail on the extent to which the age profile of Ireland’s population differs from other high-income countries. This could be presented in the Introduction, or in the Methods (setting) section.

>>>We have added a new Figure 1 comparing Ireland’s population distribution with the rest of the EU-28.  Data are from the last Eurostat Census (2011) and are added to the OSF underlying data appendices.

**R2/C5** Data sources: Please reference data sources used in the first paragraph under Data sources. 

>>>References added.

**R2/C6** Perhaps clarify what are ‘exposure’ variables where they are first mentioned.

>>>We have reworded the end of paragraph 2 in Methods>Data sources, so ‘exposure variables’ are now mentioned for the first time in their own sub-section, where they are defined.

**R2/C7** Please add content to reference 34 link – further details could not be accessed. 

>>>This reference mistakenly redirects the reader to the OSF registration, not the OSF content. We have asked editorial to correct.  We have also revised how appendices are described in the text so that readers know which specific file to open.

**R2/C8** Please check page 4, last paragraph – last sentence – should this say ICD-10 codes instead of CSO codes?

>>>Revised to:

For further details on concordance between the CSO’s use of ICD-10 codes…

**R2/C9** In analysis 2 and 3, please justify why those *living *with cancer (as opposed to those who died with cancer) can be classified as having a disease associated with palliative care need, given increased survivorship in those with a cancer diagnosis.

>>>Explanation provided in Introduction>Rationale and aim:

policy and clinical guidelines increasingly recommend palliative care across the trajectory of serious disease, meaning that those dying from these diseases are only a subset of the overall population health need (refs). To inform decision-making in policy and practice, including the targeting of interventions to those who benefit, groups cannot be defined by their characteristics at death and instead must be identified prospectively on the basis of clinical and other relevant factors (refs).

**R2/C10** Please add detail on step-by-step calculations for Analysis 2 & 3 – reference 34 contains no data.

>>>See R2/C7.

**R2/C11 **Please recheck percentages in Results, paragraph 2.  A 30-year increase of 68% is reported.  Is this correct?  Should this be 61% over 31 years? Please clarify.  

>>>We replaced the 2015 figure with the 2016 figure, and this is a 30-year change.

**R2/C12** Please provide detail on how the increase in absolute numbers of deaths from a disease associated with palliative care need was calculated (78% to 84%) as the percentages are not presented in the table. Please recheck all percentages reported. Is 84% correct?

>>>These numbers are correct and correspond to the relevant cells.  We have added more explanatory text prior to Table 2 to assist interpretation:

We quantify changing need over time in three ways in Table 2: absolute number of projected deaths with palliative care need in a given year (denoted n _y_), this absolute number as a proportion of all projected deaths in a given year (denoted p(N _y_)), and this absolute number relative to absolute number of projected deaths with palliative care need at baseline in 2016 (denoted ‘Rate of change index’).

**R2/C13** Please check first line which states that the number of people in Ireland dying from a disease associated with palliative care need is estimated to increase between 68% to 84%.   However in the results section, end of paragraph 3, the percentages mentioned are 78% to 84%.  Please clarify.

>>>As well as changes under R2/C12, we revise the opening section of the discussion as follows:

The number of people in Ireland dying from a disease associated with palliative care need is estimated to increase 68% between 2016 and 2046 if levels of need stay constant.  Under assumptions of changing need, increases are estimated in the range 78–84% depending on projection method employed

**R2/C14 **Please double-check Etkind *et al. *study for equivalent estimates for England and Wales (43% or 47% in Etkind *et al*?).

>>>Our 43% comes from the abstract (an increase of 161842 to 537240) but in their Discussion they do report 47% which can be sourced to a different projection method (which gives an increase of 175,336 to 550,734).  Since we also used this latter method, you are correct that this is a valid comparison.  43% has been changed to 47% at all points.

**R2/C15 **Please elaborate on how Ireland’s younger population might account for the greater increases in palliative care need in Ireland compared with England and Wales.  This is not totally clear given that most of the increases in both studies comes from increases in the number of people dying aged 85+

>>>Following R2/C4 above, we have added Figure 1 to illustrate this point and also this explanatory text (Methods>Setting):

Compared to the other 27 countries in the European Union (EU), Eurostat’s 2011 Census data show that Ireland has a higher proportion of its population for each age group under the age of 40.  The EU average is higher for each age group over 40.  Therefore Ireland can be seen as relatively early in the current trend for global ageing: in 20-30 years’ time it will have proportionally larger numbers moving into older age and retirement compared to other high-income countries who are already experiencing these trends 19 .  Need for geriatric and palliative care will rise accordingly as the population ages.

**R2/C16** Limitations – last paragraph – should Table 1 read Table 2?

 >>>Correct. We have changed.

**R2/C17 **“ *and that endeavours to close the policy-implementation gap*”. Is there something missing from this sentence?

 >>>Revised for clarity:

There is a growing literature recognising that policy enactment does not translate directly to policy success. Rather, implementation and continued support for the policy after enactment are essential.   

**R2/C18** As above, please recheck data (84% increase?). 

>>>Please see R2/C12 and R2/C13.

# Peer review: Reviewer response for version 1

**DOI:** 10.21956/hrbopenres.14060.r27048

**Published:** 2020-01-08

**Authors:** Staffan Lundström

**Affiliations:** 1 Department of Palliative Medicine, Stockholms Sjukhem Foundation, Stockholm, Sweden; 2 Department of Oncology-Pathology, Karolinska Institutet, Stockholm, Sweden

This paper sets out to estimate palliative care needs in Ireland from 2016 and 30 years ahead. The authors use a method for estimation recently used in a study on future palliative care needs in England and Wales, but also adds survey data to study projections of disability burden, pain prevalence and health care utilization among older people with palliative care needs. The results show a marked increase in the need for palliative care during the studied period, and it is the authors ambition to inform authorities and stakeholders in order to advance the development of palliative care services and policy.

 The paper is well written, and the limitations are well displayed and discussed. The authors point out that data for analyses 2 and 3 were available only for people aged 50+. Palliative care needs in younger groups can be large and significant, however the numbers are smaller. The paper shows an increased need for palliative care, but could elaborate further on the need for general versus specialised palliative care - where and how should resources be allocated?

 Two specific comments:

- For readers outside of Ireland it would be valuable if the authors provided information about the current number of inhabitants in Ireland in Methods/Setting.
- Details on TILDA are provided in references, but I suggest the authors in the text provide the number of participants they base analyses 2 and 3 on, namely Wave 3 (2014) and the end-of-life interviews Waves 1-5. This would facilitate the assessment of validity of the findings.

I confirm that I have read this submission and believe that I have an appropriate level of expertise to confirm that it is of an acceptable scientific standard.

# Response

**Published:** 2020-01-21

**Authors:** Peter May

**Affiliations:** Trinity College Dublin, Ireland

We are grateful to the reviewer for their report. Changes were minor.  For each reviewer comment requiring action, we use the notation [R1/C1, R1/C2, etc], where R indicates which reviewer and C numbers each comment.  We mark our responses to each comment >>>.

**R1/C1 **For readers outside of Ireland it would be valuable if the authors provided information about the current number of inhabitants in Ireland in Methods/Setting.

>>>Added to Methods>Setting:

In 2015, the baseline year for our data, the estimated population was 4.7million people with 30,127 (0.006%) recorded deaths.

**R1/C2 **Details on TILDA are provided in references, but I suggest the authors in the text provide the number of participants they base analyses 2 and 3 on, namely Wave 3 (2014) and the end-of-life interviews Waves 1-5. This would facilitate the assessment of validity of the findings.

>>>Sample sizes added to Methods>Data sources for Wave 3 (N=6,381) and end-of-life interview (N=716).
